# Ensemble Kinetic Modeling of Metabolic Networks from Dynamic Metabolic Profiles

**DOI:** 10.3390/metabo2040891

**Published:** 2012-11-12

**Authors:** Gengjie Jia, Gregory Stephanopoulos, Rudiyanto Gunawan

**Affiliations:** 1 Chemical and Pharmaceutical Engineering, Singapore-MIT Alliance, Singapore 117576; Email: jiagengjie@nus.edu.sg (G.J.); 2 Department of Chemical Engineering, Massachusetts Institute of Technology, Cambridge, MA 02139, USA; Email: gregstep@mit.edu (G.S.); 3 Institute for Chemical and Bioengineering, ETH Zurich, 8093 Zurich, Switzerland

**Keywords:** ensemble modeling, incremental identification, dynamic flux estimation, independent parameter set, generalized mass action model.

## Abstract

Kinetic modeling of metabolic pathways has important applications in metabolic engineering, but significant challenges still remain. The difficulties faced vary from finding best-fit parameters in a highly multidimensional search space to incomplete parameter identifiability. To meet some of these challenges, an ensemble modeling method is developed for characterizing a subset of kinetic parameters that give statistically equivalent goodness-of-fit to time series concentration data. The method is based on the incremental identification approach, where the parameter estimation is done in a step-wise manner. Numerical efficacy is achieved by reducing the dimensionality of parameter space and using efficient random parameter exploration algorithms. The shift toward using model ensembles, instead of the traditional “best-fit” models, is necessary to directly account for model uncertainty during the application of such models. The performance of the ensemble modeling approach has been demonstrated in the modeling of a generic branched pathway and the trehalose pathway in *Saccharomyces cerevisiae* using generalized mass action (GMA) kinetics.

## 1. Introduction

Mathematical modeling is one of the cornerstones of metabolic engineering [1]. These models vary in their formulation and complexity depending on the specific applications. For example, flux balance analysis relies on algebraic models of metabolic networks to predict the impact of pathway perturbations (e.g. gene knock-out/knock-in) on the steady-state metabolic flux distribution [2,3]. Meanwhile, kinetic ordinary differential equation (ODE) models have been traditionally used for dynamic optimization of culture conditions in a bioreactor [4]. Regardless of the type of the models, the process of model building is typically iterative, combining wet-lab experiments and *in silico* analysis and optimization [5]. Despite much progress in both experimental and computational fronts, e.g. increasing availability of high quality and system-level data and development of efficient parameter estimation methods, the process of creating mathematical models from biological data is still very challenging [6]. Much of the difficulty of this process, especially for kinetic ODE models, is rooted in the fundamental issue of model identifiability [7], wherein it is not possible to uniquely determine model equations and parameter values from experimental data. As we and many others have shown [8,9,10,11], the estimation of unknown parameters by fitting model simulations to biological measurements is typically ill-posed. Consequently, even when the best-fit parameters are obtained, the corresponding model may have little predictive capability; or worse, it could be misleading.

The majority of existing parameter estimation methods for the kinetic modeling of metabolic networks involve a single-step estimation, in which unknown parameters are estimated simultaneously by minimizing model prediction error [6,12,13]. There are a few reasons why such a strategy is often inefficient. Kinetic models of metabolic pathways (or cellular networks in general) typically possess a large number of unknown kinetic parameters, where in some cases, the number of parameters increases combinatorially with the number of metabolites. The large number of unknown parameters means not only that the parameter estimation will involve a vast parameter search space, but also that the parameters may not even be completely identifiable from data. The first effect leads to a large-scale, often numerically intractable, global optimization problem. The latter and arguably the more important consequence implies that the estimation problem has no unique solution (*i.e.* it is ill-posed) and many parameter combinations can fit the data equally well. Multiplicity of solutions to the parameter estimation of kinetic ODE models has been documented in different biological systems [11,14].

The aforementioned issues give the motivation for developing and applying a different framework to construct metabolic and biological models from data, one that can explicitly account for model uncertainty. In this work, an ensemble modeling strategy is employed. Ensemble modeling has previously been applied to address structural uncertainty in the modeling of metabolic and other biological networks. For example, ensemble models of metabolic pathways could be created by enforcing thermodynamic feasibility constraints on the metabolic reactions and used for metabolic control analysis [15,16,17,18]. In a modeling study of TOR (target of rapamycin) signaling pathway in yeast, an ensemble of 19 kinetic ODE models was generated, where each model in the ensemble represented a different hypothetical topology of the pathway [19]. The process of creating an ensemble of models from the set of possible components and reactions in a biological network has also recently been automated [20]. In these studies, a comparative analysis of models in the ensemble was conducted to determine the most likely mechanistic explanation for some experimental observations. For nonlinear discrete time dynamic system, an ensemble modeling approach has also been proposed using the set membership framework, without requiring any prior assumption on the functional form of the model equations [21]. 

Here, we describe a step-wise model identification approach for the creation of an ensemble of kinetic ODE models from metabolic time profiles. Unlike the ensemble modeling work mentioned above, this approach is applied to tackle the uncertainty in the estimation of kinetic parameters. That is, models in the ensemble will share the same network topology, but differ in their parameter values. In essence, these models represent regions in the parameter space from which model prediction errors are (statistically) equivalent. Such an ensemble can be generated by exploring the parameter space using existing methods such as Metropolis-type random walk Markov chain [22] and the Pareto Optimal Ensemble Techniques (POETs), the last of which is based on multi-objective optimization [14]. However, the search was done over the full parameter set in these techniques, and thus the computational requirement may increase quickly with the number of kinetic parameters. In this work, a new and numerically efficient ensemble modeling procedure is developed based on the incremental identification or dynamic flux estimation (DFE) [23,24] and employing an adaptive efficient Metropolis Monte Carlo sampling [25]. The performance of the ensemble modeling procedure has been demonstrated using models of a generic branched metabolic pathway [26] and the trehalose pathway in *Saccharomyces cerevisiae* [27,28].

## 2. Ensemble Kinetic Modeling

Ordinary differential equations have been commonly used to model metabolic pathways. The model equations describe the mole balance around metabolites as they are enzymatically transformed from one to another. In this case, the system is assumed to be well-mixed (*i.e.* ignoring spatial distribution of metabolites) [29], leading to the following general form:

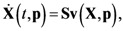
(1)
where *t* denotes the time, **p** is the parameter vector, **X**(*t*,**p**) is the vector of *m* metabolite concentrations, **v**(**X**,**p**) denotes the vector of *n* enzymatic reactions/fluxes, and **S** is the *m×n* stoichiometric matrix. The metabolic fluxes are further specified as the function of **X**, for example using a power-law dependence:

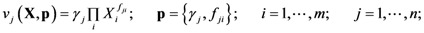
(2)
where the parameter *γ_j_* is the rate constant of the *j*-th flux and *f_ji_* is the kinetic order, reflecting the influence of metabolite *X_i_* on the *j*-th flux (positive: substrate or activation, negative: inhibition). Aside from the power-law function, Michaelis-Menten and Hill equations have also been commonly used to describe the kinetics of enzymatic reactions **v**(**X**,**p**).

The aforementioned power-law model, also known as generalized mass action (GMA) model, belongs to a widely adopted framework for the modeling and analysis of biochemical processes, the Biochemical Systems Theory (BST) [29,30,31]. Power-law models have a relatively simple structure that permits algebraic manipulation in logarithmic scale. Furthermore, the nature of network connectivity is directly related with the parameter values of rate constants and kinetic orders, facilitating simultaneous parameter estimation and network structure identification. However, the estimation of parameters of these models is known to be very challenging, even after the development of over 100 methods [32]. Perhaps this difficulty is not surprising, as the number of parameters in power-law models is often large and this number increases quickly with network complexity (*i.e.* the number of metabolites and interactions). Consequently, the parameters are typically not completely identifiable [10], motivating the application of the ensemble modeling developed in this work. 

Briefly, the proposed ensemble modeling derives from the incremental identification or dynamic flux estimation method [24,33]. In these methods, the estimation of unknown kinetic parameters from concentration time profiles **X***_M_* (*t*) is decomposed into a few steps, involving (1) the computation of slopes of time-series data **Ẋ***_M_* (*t*), (2) the calculation of dynamic flux profiles **v**(*t*) from **Ẋ***_M_* (*t*), and finally (3) the regression of parameters, which can be done one flux at a time. In the original formulation of the incremental identification and DFE, the number of measured species is assumed to be larger than the number of reactions, such that the second step possesses a unique solution. However, since metabolic pathways typically involve more fluxes than metabolites, there now exist (infinitely) many dynamic flux values, each of which is a mathematically valid solution. This is the premise of the new ensemble modeling method. Specifically, models in the ensemble represent a subset of the dynamic flux solutions to **Ẋ***_M_* = **Sv**, with additional criteria that the kinetic parameters produce statistically equivalent and biologically relevant model predictions. The construction of the model ensemble is detailed in the Method section.

### 2.1. A Generic Branched Pathway

**Figure 1:**
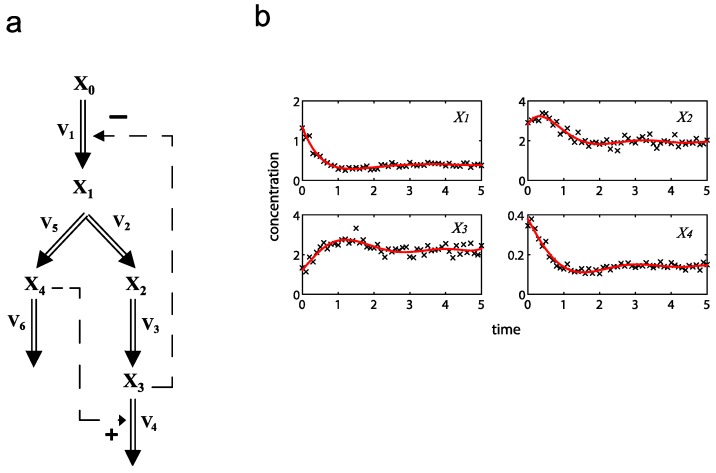
A generic branched pathway. (**a**) Metabolic pathway map. Metabolic fluxes: double-line arrows, regulatory interactions: dashed arrows with signs; (**b**) The smoothened data (red line) *versus* the noisy data (**×**).

The metabolic pathway map in this case study is given in Figure 1a, which describes the transformations among four metabolites with both feedback activation and inhibition. The model of the pathway is written as a GMA model with 13 kinetic parameters, as shown in Equation (3). This model with the reported parameter values (see Table S1 in Supplementary Material) and initial concentrations [26] was used to generate time-course concentration data, contaminated with i.i.d. Gaussian noise with zero mean and 10% coefficient of variation (the ratio of standard deviation to the mean). For validation purpose, two independent datasets were generated in the same manner as above, but with different initial conditions [*X*_1_(*t*_0_)  *X*_2_(*t*_0_)  *X*_3_(*t*_0_)  *X*_4_(*t*_0_)] = [4  1  3  4] and [0.2  0.3  4.2  0.01], respectively. The *in silico* noisy data were smoothened using a *6*-th order polynomial, which gave the best polynomial fit to the data according to adjusted R^2^ [34] and Akaike Information Criterion (AIC) [35] (see Figure 1b). Subsequently, a central finite difference approximation was applied to compute the time-slopes of the smoothened data.

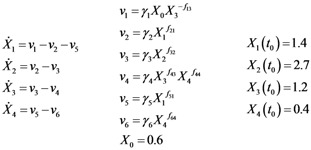
(3)


In this example, the degree of freedoms is 2 (4 metabolites and 6 fluxes). Fluxes *v_1_* and *v_6_* were chosen as the independent fluxes, since this selection led to an invertible **S***_D_* and comprised the least number of independent parameters. The involved independent parameters **p***_I_* included the rate constants {*γ_1_*, *γ_6_*} and the kinetic orders {*f_13_*, *f_64_*}, which were constrained to within [0, 100] and [0, 5], respectively. The bounds for dependent parameters were set to be the same, *i.e.* {*γ*_2_, *γ*_3_, *γ*_4_, *γ*_5_} ∈ [0,100], {*f*_21_, *f*_33_, *f*_43_, *f*_44_, *f*_51_} ∈ [0,5]. In addition, the upper bound for allowable metabolic fluxes in this artificial network was set as 5×10^5^ mM/min. 

**Figure 2:**
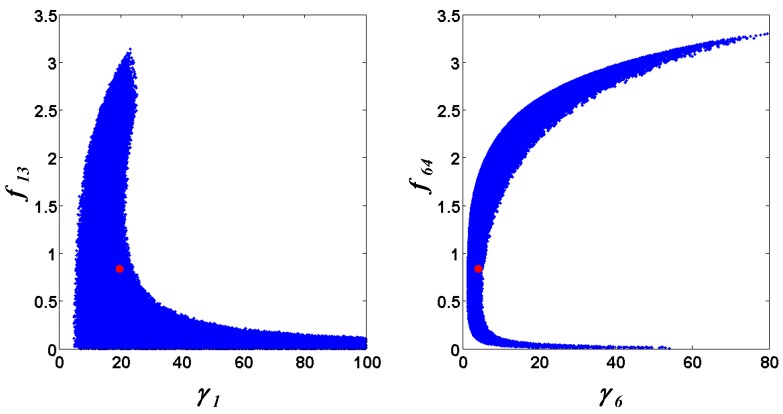
Two-dimensional projections of the viable parameter space onto the parameter axes of each independent flux (*v_1_*: left, *v_6_*: right). The true parameters are marked in red.

**Figure 3:**
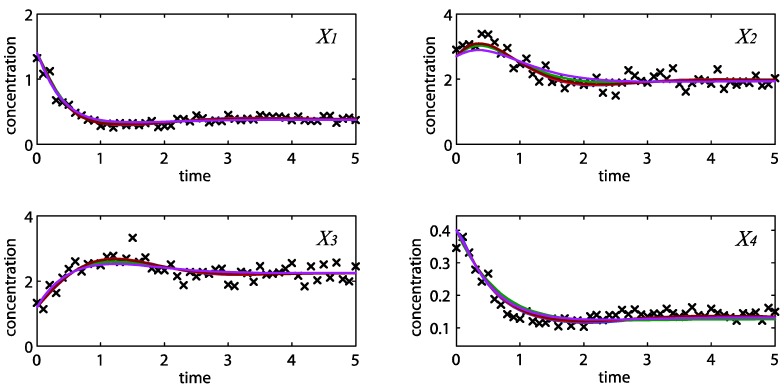
Concentration simulations of five randomly selected models from the ensemble (solid blue, brown, green, red and purple lines) *versus* the noisy data (**×**).

**Figure 4:**
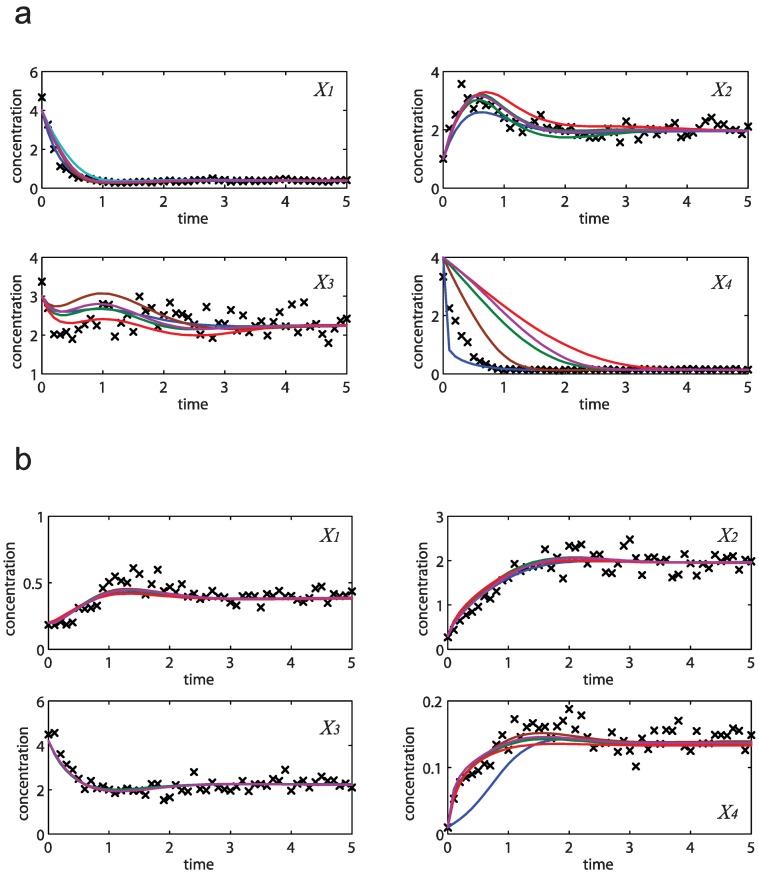
Concentration simulations of the same five models as in Figure 3 (solid blue, brown, green, red and purple lines) *versus* independent datasets (**×**), with initial concentrations of [4 1 3 4] (**a**) and [0.2 0.3 4.2 0.01] (**b**).

Following the ensemble modeling procedure described in the Method section, the initial parameter point for the out-of-equilibrium adaptive Metropolis Monte Carlo (OEAMC) algorithm was taken from the parameter estimation minimizing the flux error function Φ*_R_* (minimum 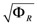 = 0.130), and the upper 95% confidence bound of the error function value was determined using Monte Carlo approach (viable 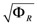 < 0.347). Table 1 summarizes the outcome of the ensemble modeling. The multiple ellipsoid-based sampling (MEBS) algorithm produces a model ensemble with 59,928 members within the viable parameter subspace. The corresponding volume of the viable subspace represented only 0.284% of the original parameter space (*i.e.* the space defined by the upper and lower parameter bounds). Figure 2 shows the projections of the viable regions onto the two-dimensional parameter axes of each independent flux. The true parameter values are contained in the viable subspace, and thus belong to the ensemble (red dot in Figure 2). The member models of the ensemble were able to predict the concentration and slope profiles reasonably well (see Table 1), even when the ensemble was constructed using a different error function. The comparison of data and model predictions in Figure 3 demonstrates the equivalence among five randomly selected models in the ensemble. Finally, Figure 4 shows the comparison of model simulations from the same five models and independent (simulated) experimental datasets, indicating that these models could predict the systems dynamics under different initial conditions reasonably well. 

**Table 1:** Ensemble kinetic modeling of the branched pathway model using Φ*_R_*.

CPU time (sec) ^a^	1664
Calculated volume of initial parameter space (*V_ci_*) ^b^	2.5 × 10^5^
Estimated volume of viable parameter space (*V_ev_*) ^c^	710.1 ± 5.1
Ratio of *V_ev_* to *V_ci_*	(284.0 ± 2.0) × 10^−3^%
Range of slope errors 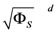	[1.370 × 10^−1^, 5.081 × 10^−1^]
Range of concentration errors 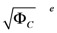	[3.554 × 10^−2^, 2.150 × 10^−1^]

a. The CPU time was the total time for the ensemble construction, which was run on a computer workstation with Dual Processors Intel Quad-Core 2.83 GHz. b. *V_ci_* was calculated by simple multiplications of the independent parameter ranges. c. *V_ev_* was calculated by integrating the volumes of an ensemble of ellipsoids that cover the viable parameter space [25]. d. The range of slope error was computed using Equation (14) for all models in the ensemble. e. The range of concentration error was computed by Equation (15) for all models in the ensemble.

Note that besides the Φ*_R_* minimization, the proposed kinetic ensemble modeling approach can also use other error functions. The viable parameter space using the slope error Φ*_S_*, for example, closely resembles that shown in Figure 2 (see Supplementary Material), demonstrating the robustness of the procedure in capturing the model uncertainty.

### 2.2. The Trehalose Pathway in Saccharomyces cerevisiae

**Figure 5:**
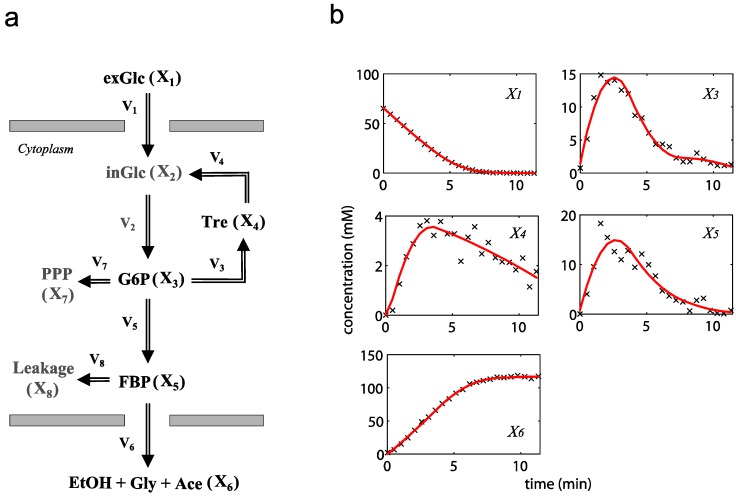
The trehalose pathway in *Saccharomyces cerevisiae*. (**a**) Metabolic pathway map. Metabolic fluxes: double-line arrows; (**b**) The smoothened data (red line) *versus* the noisy data (**×**).

The second case study was taken from the modeling of the glycolysis and trehalose production in the baker’s yeast *Saccharomyces cerevisiae*. Figure 5a shows the metabolic pathway and Equation (4) presents the GMA model, describing in a simplified fashion how glucose is converted into trehalose and other products in a cyclic pathway [28]. The notations for the concentrations of metabolites are as follows: extracellular glucose (exGlc) – *X_1_*, intracellular glucose (inGlc) – *X_2_*, glucose 6-phosphate (G6P) – *X_3_*, trehalose (Tre) - *X_4_*, fructose 1, 6-biphosphate (FBP) – *X_5_*, extracellular end-products (ethanol, glycerol and acetate) – *X_6_*, pentose phosphate pathway (PPP) – *X_7_* and other pathways (Leakage) – *X_8_*. The variables *V_ex_* and *V_in_* denote the extracellular (5.00×10^−2^ L) and intracellular (7.17×10^−3^ L) volumes of the bioreactor and the cell population, respectively. The time-course concentration data have been obtained using *in vivo* NMR, but only *X_1_*, *X_3_*, *X_4_*, *X_5_* and *X_6_* were measured [27]. In the following, we used the dataset from normally grown cells at 30 °C that were fed with a pulse of glucose. The raw experimental data were smoothened using a piecewise cubic spline, the fitting of which was validated by adjusted R^2^ [34] and AIC [35] (see Figure 5b). Like before, a central difference approximation was applied to obtain the time-slopes of concentration data.

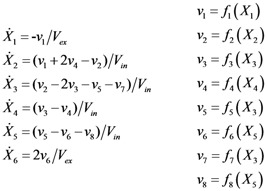
(4)


The original ODE model contains 6 metabolites and 8 fluxes, as shown in Equation (4). In this case study, the ODE for *X_7_* and *X_8_* are removed, as their concentrations do not affect the other metabolites (*i.e.* they are sinks in the system). While the intracellular glucose *X_2_* was not measured, its rate of change can be obtained from the measured metabolites by performing an overall mass balance around the pathway, resulting in the following relationship:

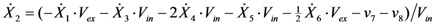
(5)
Using this relationship, the model can be reduced to the following equations:

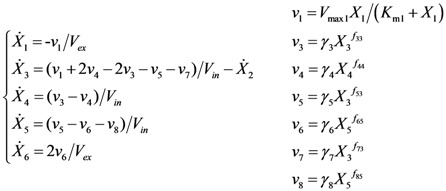
(6)


According to Equation (4), we have 3 degrees of freedom (5 measured metabolites and 8 fluxes). Here, fluxes *v_4_*, *v_7_* and *v_8_* were chosen as the independent fluxes, by the same rationale as before. Correspondingly, the independent parameters **p***_I_* comprised the rate constants {*γ*_4_, *γ*_7_, *γ*_8_} and the kinetic orders {*f*_44_, *f*_73_, *f*_85_}, which were constrained within [0, 100] and [0, 5], respectively. Note that the glucose transport flux (*v_1_*) was modeled using Michaelis-Menten (MM) kinetics instead of the power law, as this was found to be a better fit to the time profile of *X*_1_ (a constant decrease at high *X*_1_ and an exponential-like time profile at low *X*_1_). The regression of the MM kinetic parameters can also be casted as a linear regression problem as follows:

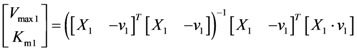
(7)
where [*X*_1_ ∙ *v*_1_] is the vector of element-wise multiplication of *X*_1_ and *v*_1_. Finally, the upper bound for flux values was set as 5 × 10^5^ mM/min, according to the maximal flux value reported in a similar glycolytic pathway [36]. 

**Figure 6:**
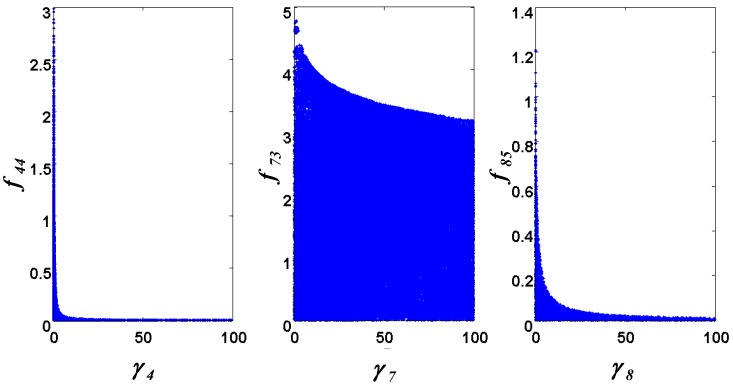
Two-dimensional projections of the viable parameter space onto the parameter axes of each independent flux (*v_4_*: left, *v_7_*: middle, *v_8_*: right).

**Figure 7:**
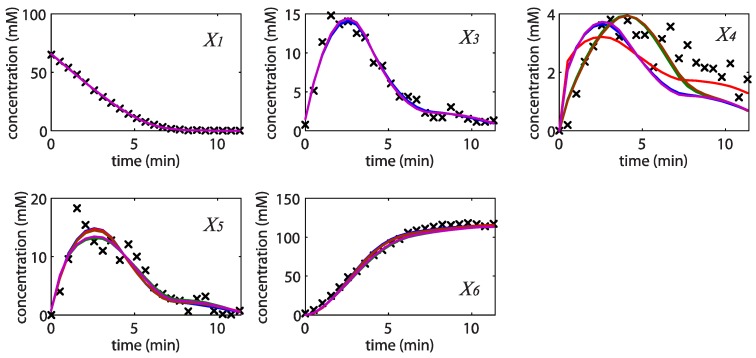
Concentration simulations of five randomly selected models from the ensemble (solid blue, brown, green, red and purple lines) *versus* the experimental data (×).

The initial parameter point for the OEAMC algorithm was again obtained by minimizing Φ*_R_* (minimum 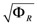 = 7.64 × 10^−2^) and the upper 95% confidence bound was found using a Monte Carlo approach (viable 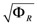 < 0.186). Table 2 gives the summary of the model ensemble for the trehalose model. The model ensemble was represented by 3423 member models, and the volume of the corresponding viable subspace constitutes 2.59 × 10^−3^% of the original constrained parameter space. The slope errors were acceptable, but the concentration errors had a high upper bound. Upon a closer inspection, only a minority of the model (3 out of 3423) had concentration errors larger than 10^2^, and removing these, the upper bound for the concentration error reduces to 35.92. This issue is not unexpected as the model ensemble was created based on the flux error function and not the concentration error. In particular, there is no guarantee that parameter values with a small flux error will also provide a low concentration error. However, we note that the divergence between the flux error and concentration error functions occurred only rarely (< 0.1%). Figure 6 shows the projections of the viable parameter subspace onto the two-dimensional parameter axes of each independent flux. Finally, Figure 7 shows a comparison between the concentration predictions of five randomly chosen models from the ensemble and the measured metabolite time profiles, again demonstrating that models in the ensemble can reproduce the data equally well.

**Table 2:** Ensemble kinetic modeling of the trehalose pathway model using Φ*_R_*.

CPU time (sec)	6489
Calculated volume of initial parameter space (*V_ci_*)	1.25 × 10^8^
Estimated volume of viable parameter space (*V_ev_*)	3237 ± 125
Ratio of *V_ev_* to *V_ci_*	(25.90 ± 1.00) × 10^−4^%
Range of slope errors 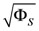	[5.825, 46.42]
Range of concentration errors 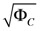	[1.125, 3.880 × 10^2^]

## 3. Discussion

The difficulty in simultaneously estimating kinetic parameters of metabolic models is often caused by a lack of complete parameter identifiability [10]. In other words, not all parameters can be uniquely identified and many parameter combinations can give similar goodness-of-fit to the available data [11]. Hence, even when the parameter estimation algorithm could return best-fit values, the resulting model may have little predictive capability; or worse, could be misleading. In the present work, a different approach is taken that directly addresses the issue of model uncertainty through the generation of an ensemble of models. The member models are equivalent in the sense that (1) the models closely approximate the same mass balance equation and (2) the model approximations are statistically equal (to within a 95% confidence level). Although the case studies mainly involved GMA models with power-law flux functions, the ensemble modeling procedure can be used for any form of flux functions, as long as the ODE model follows Equation (1). For power-law and Michaelis-Menten kinetics, the least square regression of the dependent parameters reduces to linear regression, and thus can be done very efficiently. The main reason to use power-law models here was that they represent some of the most challenging problems in kinetic modeling due to the large parameter space, the lack of complete parameter identifiability, stiff ODEs and high degree of nonlinearity. 

In this work, we have used the DOF in estimating dynamic fluxes from time-slopes of concentration data **Ẋ** (*t_k_*) = **Sv** (*t_k_*), to restrict the parameter subspace within which the model ensemble is created. Since this DOF is associated with the stoichiometric matrix **S,** the same ambiguity also exists, albeit implicitly, when the original ODE model: **Ẋ** (*t*) = **Sv** (*t*) is integrated during the parameter estimation. In corollary, there can exist more than one **v**(*t*) that agree with the same **X**(*t_k_*). However, in this case, the calculation of **v***_D_*(*t*) will involve an infinite dimensional vector space (function space). Furthermore, we note that the ambiguity mentioned above is different from the parametric uncertainty that is represented by the ensemble modeling. In particular, the equivalency of models in the ensemble is judged by the error function Φ and different error functions can produce dissimilar model ensembles. As shown in the second case study, a few models of the ensemble created by Φ*_R_* produced large concentration errors Φ*_C_*. This discrepancy is perhaps not surprising as Φ*_R_* is based on the algebraic model **Ẋ** (*t_k_*) = **Sv** (**X** (*t_k_*), **p**), while the calculation of Φ*_C_* involves the integration of the ODE model **Ẋ** (*t*) = **Sv** (**X** (*t*), **p**)..

The proposed ensemble modeling method has the advantages that (1) the model ensemble is compactly defined using a small number of independent parameters; (2) the dependent parameters can be efficiently computed from the independent parameters; (3) only biologically-meaningful models are included in the model ensemble; and (4) data uncertainty (noise) is explicitly accounted for. The first two aspects come as courtesy of the step-wise identification approach adopted in the development of the method. The computational cost of constructing the model ensemble is related with the parameter exploration and the computation of the error function. The compactness of the parameter space of the ensemble is therefore particularly important for numerical efficiency and ultimately for practical applications. For OEAMC and MEBS algorithms, the number of required parameter samples during parameter exploration has been shown to increase linearly with the parameter dimension, which in this case is equal to the number of independent parameters [25]. On the other hand, the computational cost of a single evaluation of the error function primarily comes from the least square regression of the dependent parameters and possibly from the integration of the ODE, if the error function requires the simulation of **X**(*t*). For the error function used in the case studies above, this computational cost should increase linearly with the number of dependent fluxes, assuming that the number of unknown parameters in each dependent flux stays about the same. 

In the proposed ensemble modeling, the model uncertainty is related to parametric uncertainty that arises from data noise, leaving out the contribution of structural uncertainty (mismatch between the assumed model equations and the true dynamics). Increasing data noise is therefore expected to increase the size of the model ensemble, *i.e.* the volume of the viable parameter subspace, by directly changing the statistics of the error function. However, in this case, higher noise in data will also lead to more uncertainty in the time slopes estimates of the concentration data. Since the direct (error function) and indirect (smoothing and slope calculation) effects of data noise could not be easily separated, we have chosen a Monte Carlo approach in determining the confidence bound of the error function (see Method section). 

We have also made the assumption that there exists a unique solution to the computation of **p***_D_* from **p***_I_*. For GMA models, this assumption requires that (1) the number of time points exceed the number of parameters **p***_D_* from each flux (not the total number) and (2) the logarithm of the metabolite concentration time profiles appearing in each flux are linearly independent. The first requirement is usually satisfied as the number of parameters involved in every flux ranges only between 2 and 5. The second requirement depends on the experimental conditions, but is again usually fulfilled since each flux depends only on a handful of metabolites and data are contaminated with random noise. If this assumption becomes invalid for one or more dependent fluxes, then these fluxes can be included into the set of independent fluxes, at the cost of increasing the dimensionality and computational time of the parameter exploration step. In such a case, the calculation of dependent fluxes from the independent flux values will require taking a pseudo-inverse of **S***_D_* (see Method).

Constraints on parameters and fluxes are important in restricting the size of the ensemble, in a problem dependent manner. For example, in the first case study, the ensemble hit the lower constraints on both kinetic order parameters (set at 0) and the upper constraint for the rate constant *γ*_1_ (see Figure 2). Meanwhile, parameter constraints affect the second case study more than the first, where the lower and upper constraints of all rate constants and the lower bounds of all kinetic orders limited the viable parameter subspace (see Figure 6). Furthermore, in both case studies, the requirement for positivity of the flux values (*i.e.* lower bounds of the fluxes) was an important constraint, as this was frequently violated during the parameter exploration (data not shown). 

The ensemble modeling can be integrated into the iterative model building procedures for biological systems [6]. In this case, the ensemble size will be reduced after every iteration, by removing member models that are not consistent with (additional) time-series concentration data from new experiments. The ensemble of models can also be pruned using steady-state data from knock-out studies and/or thermodynamic constraints [16]. In addition, the benefits of improving the quantification of dynamic fluxes will immediately materialize as such data can be directly used in the proposed method. 

Finally, the ability to generate an ensemble of kinetic models also necessitates the development of new methodologies on how to utilize such ensemble. The obvious challenge is how to analyze and/or optimize the system when it is represented by a set of models, not just one model, possibly containing a large number of members. Here, we suggest two strategies: the first involves the generation of a (random) sample of models from the ensemble and in such a case, the results from the analysis and optimization can be represented in the form of a histogram. The second strategy is to take the advantage that the ensemble model generation involves only linear (or log-linear) algebraic equations. In this case, interval or constraint propagation using interval arithmetic can be used to evaluate upper and lower bounds for the system behavior, as done previously for GMA models [37]. 

## 4. Method

### 4.1. Problem Formulation

The ensemble modeling procedure is based on the incremental identification or DFE approach for parameter estimation, where kinetic parameters are estimated in three incremental steps. Initially, given time-course concentration measurements **X***_M_* (*t_k_*), *k* = 1, *K*, the estimation procedure starts with the computation of time-slopes. Data smoothing is usually applied to improve the numerical estimation of **Ẋ***_M_* (*t_k_*). The slopes can be estimated using a finite difference approximation of the smoothened data or by differentiating the smoothened curve function, if available. Subsequently, the values of dynamic reaction fluxes are approximated from the mass balance **Ẋ***_M_* (*t_k_*) = **Sv**(*t_k_*). Finally, the kinetic parameters are determined from dynamic flux values using a least square regression **v**(*t_k_*) = **v**(**X***_M_* (*t_k_*), **p**) which can now be done for each flux individually. By decomposing the identification problem into smaller easy-to-do subproblems, the step-wise identification can offer a significant reduction in the computational cost of performing the estimation. Furthermore, for power-law flux functions, the third step involve only simple (log-)linear regressions. However, in the original formulation of incremental identification and DFE, one assumes that the subproblems have a unique solution, which is often invalid for a metabolic pathway. 

Consider the typical scenario where the number of reactions in the metabolic pathway exceeds that of metabolites (*i.e.*, *m < n*). In this case, there theoretically exist an infinite number of dynamic flux **v**(*t_k_*) that can satisfy the mass balance equation **Ẋ***_M_* (*t_k_*) = **Sv** (*t_k_*), each of which represents a valid mathematical solution to the parameter estimation problem. The dimensionality of the dynamic flux solutions is equal to the degree of freedom (DOF) in the mass balance, defined as the difference between the number of fluxes and the number of metabolites: *n_DOF_* = *n−m >* 0. Thus, only a subset of *n_DOF_* fluxes (called independent fluxes) need to be specified at each time point *t_k_*, while the remaining (dependent) fluxes can be computed from the mass balance equation. 

In the following, the flux vector is decomposed into **v**(*t_k_*) = [ **v***_I_*(*t_k_*)*^T^* **v***_D_*(*t_k_*)*^T^* ] *^T^*, where the subscripts *I* and *D* denote the independent and dependent subsets, respectively. Similarly, **S** and **p** are restructured as **S** = [ **S***_I_* **S***_D_* ] and **p** = [ **p***_I_* **p***_D_* ]. As mentioned above, given the values of **v***_I_*(*t_k_*), one can compute the corresponding values of **v***_D_*(*t_k_*), according to:

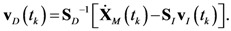
(8)
Assuming that **S** has a full row rank, one can choose *n_DOF_* independent fluxes such that **S***_D_* is invertible. For numerical efficiency, the independent fluxes are chosen by considering the following: (i) the **S***_D_* is invertible, (ii) the number of the independent parameters **p***_I_* is small, and/or (iii) **p***_I_* values are known *a priori* within a small range. Similar numerical considerations for selecting flux functions have also been discussed elsewhere [38]. Subsequently, by replacing **v***_I_*(*t_k_*) with the flux function **v***_I_*(**X***_M_*(*t_k_*),**p***_I_*) and assuming that the dependent parameters **p***_D_* can be uniquely determined from **v***_D_*(*t_k_*), then the model parameters can be completely defined by assigning the values of the independent parameters **p***_I_*. For power-law models, the uniqueness of **p***_D_* is a weak assumption, requiring the least square regression problem **v***_D_* (*t_k_*) = **v***_D_* (**X***_M_* (*t_k_*), **p***_D_*) to be fully or over-determined (see Discussion section). 

In the above, we have assumed that time-series data for all metabolites in the model are available. When one or more metabolites are not measured, we can modify the procedure by first rewriting the ODE model, separating the balances associated with those that are measured and those that are not:

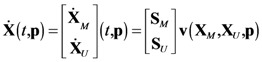
(9)
where the subscripts *M* and *U* refer to the measured and unmeasured metabolites, respectively. The independent fluxes are then selected such that the dependent fluxes can be computed using the following relationship:

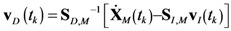
(10)
where **S***_I,M_* and **S***_D,M_* are submatrices of **S***_M_*, such that **S***_M_* = [ **S***_I,M_* **S***_D,M_* ] following the decomposition of **v**(*t_k_*) = [ **v***_I_^T^* **v***_D_^T^* ]*^T^*. As expected, the degree of freedoms will increase (*n_DOF_* = n−m*, where m* is the number of measured metabolites), and so will the number of independent fluxes. The independent fluxes should be selected such that **S***_D,M_* is invertible and should also include fluxes that appear in **Ẋ***_U_*. The same practical considerations for choosing **v***_I_*, e.g. considering the number of and the prior information on **p***_I_*, are also applicable. Finally, like before, given the values of **p***_I_*, the dependent parameters can be obtained by least square regression of **v***_D_*(*t_k_*). However, since **v***_I_*(*t_k_*) can also depend on **X***_U_*, *i.e.* **v***_I_*(**X***_M_*(*t_k_*), **X***_U_*(*t_k_*), **p***_I_*), we will need to simulate **Ẋ***_U_* = **S***_U_***v**(**X***_M_*, **X***_U_*, **p**), using the smoothened **X***_M_*(*t*) as input variables. 

Here, the model ensemble embodies two types of uncertainty: mathematical and statistical. The mathematical uncertainty is related to the aforementioned DOF in the mass balance, while statistical uncertainty is associated with noise in the concentration data. Now, even when different combinations of **p***_I_* and **p*_D_*** are obtained from the relationship **Ẋ***_M_*(*t_k_*) = **Sv**(*t_k_*), they may not give the same goodness-of-fit to the concentration measurements **X***_M_*(*t_k_*). Briefly, the difference in the quality of data fitting is due to the fact that the mathematical equivalence above is established based on the slopes of the (smoothened) concentration data, not on the concentrations themselves, and also due to noise in data. Here, the ensemble modeling is performed by exploring the parameter space **p***_I_* and demarcating the viable subset of parameters that satisfy both **Ẋ***_M_*(*t_k_*) = **Sv**(*t_k_*) and two additional criteria: (1) all kinetic parameter values and fluxes are within biologically relevant bounds and (2) the model prediction error is within acceptable statistical bounds. Details of the parameter exploration algorithm and parameter viability criteria are given below.

### 4.2. HYPERSPACE Toolbox

**Figure 8:**
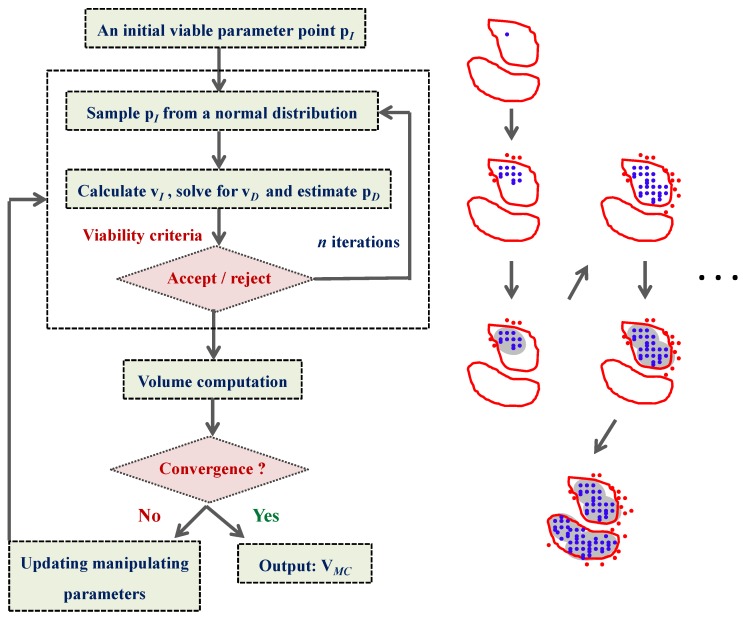
Flowchart of the out-of-equilibrium adaptive Metropolis Monte Carlo (OEAMC) algorithm. On the right, the red closed curves represent hypothetical contour plots of the viable parameter space. The viable points are marked in blue and the nonviable points are marked in red. Finally, the grey areas illustrate the minimum volume enclosing ellipsoids. This figure is adapted from the original publication [25].

In this work, the parameter exploration is carried out using the HYPERSPACE toolbox, specifically the out-of-equilibrium adaptive Metropolis Monte Carlo (OEAMC) and multiple ellipsoid-based sampling (MEBS) method [25]. These methods have been shown to be effective in exploring high-dimensional, non-convex and poorly connected viable spaces. Briefly, the OEAMC method provides a coarse-grained global exploration of the viable parameter space. The resulting coarse-grained set in turn becomes the starting point for a fine-grained local exploration offered by the MEBS to further characterize the viable parameter space. The OEAMC algorithm was developed from a combination of Metropolis Monte Carlo sampling [39] and Simulated Annealing [40]. Given an initial viable parameter point, the OEAMC carries out *n* iterations in which new parameter points are sampled from a normal distribution and subjected to the viability criteria. After every *n* iterations, the algorithm determines whether the sampling should be continued depending on a convergence condition. In this case, the viable parameters (blue dots in Figure 8) found so far are grouped into hyper-ellipsoids of minimum volume (grey areas in Figure 8), which are constructed to enclose the viable points in each cluster. The stopping criterion is then determined from the convergence of the sum of the volumes of these hyper-ellipsoids. Finally, the output from the OEAMC is the set V*_MC_* containing coarse-grained viable parameter points. Figure 8 illustrates the procedure of this algorithm.

**Figure 9:**
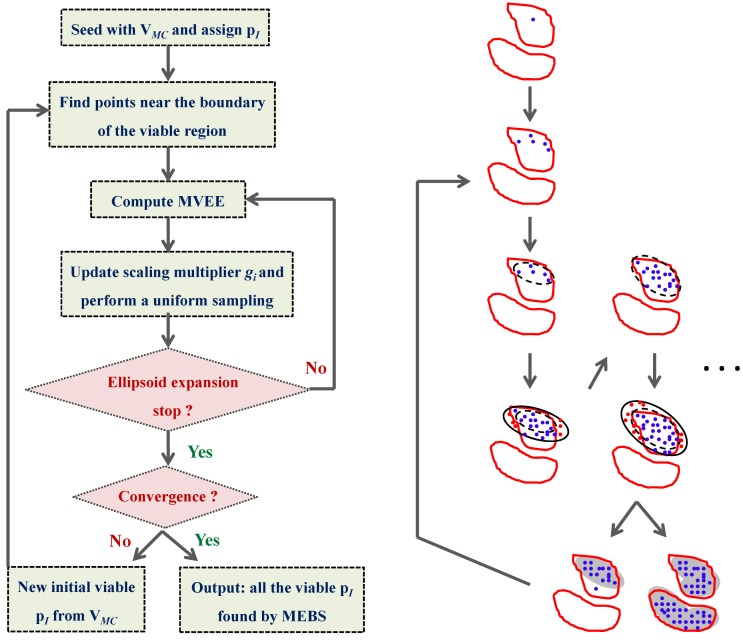
Flowchart of the multiple ellipsoid-based sampling (MEBS) algorithm. In the right part of the figure, the red closed curves represent hypothetical contour plots of the viable parameter space defined by some criteria. The viable points are marked in blue and the nonviable points are marked in red. Finally, the grey areas illustrate the minimum volume enclosing ellipsoids. This figure is adapted from the original publication [25].

The MEBS method is designed to produce fine-tuned hyper-ellipsoids that tightly bound viable regions in the parameter search space, based on another algorithm that has been introduced elsewhere [41]. The ellipsoids’ centers, orientations and lengths of axes can be adjusted in order to enclose the viable parameter regions as tightly as possible. Starting with one parameter point of the V*_MC_* set, the MEBS searches for viable parameter points near the boundary of the viable region. A Minimum Volume Enclosing Ellipsoid (MVEE, dashed ellipsoids in Figure 9) is then created to cover the local viable region. Subsequently, the MVEE is scaled up by a multiplier *g_i_* (solid curves in Figure 9), and a uniform sample of points is generated inside this scaled ellipsoid. Among these random points, the nonviable points (red points) are discarded, and another iteration of MVEE and another uniform sampling using a new multiplier *g_i+1_* are done using the remaining viable ones (blue points). The performance of the algorithm depends strongly on the multiplier *g_i_*, and here we have used the recommended scaling parameters in the original publication [25]. The iteration is repeated until the scaling multiplier tends to one or a fixed number of iterations is reached. Finally, the whole procedure above is repeated for another viable parameter point from V*_MC_* until all parameter points in this set are exhausted. The output of the MEBS is a comprehensive set of viable parameter points. Figure 9 summarizes the procedure of the MEBS algorithm.

### 4.3. Model Viability Criteria

Given any values of the independent parameters **p***_I_*, the corresponding dependent fluxes and parameters may not necessarily be biologically relevant, for example the dependent fluxes may become negative or the parameters may assume unrealistic values. Thus, in the ensemble modeling procedure, these cases are excluded by enforcing constraints on the values of fluxes and parameters, as follow:

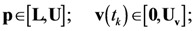
(11)
where **L** and **U** denote the lower and upper bounds for the parameters, and **U_v_** is the maximum value of metabolic fluxes. The second viability criterion is meant to establish equivalence among the member models in terms of their goodness of fit to data. If one makes the assumption that data noise comes from a Gaussian distribution, then the confidence bound of error function Φ can usually be estimated using standard statistical analyses and model sensitivities [42]. When data noise is not Gaussian, the confidence bounds can be estimated using a Monte Carlo approach [43].

In the case studies, the upper confidence bound of the error function Φ was obtained using a Monte Carlo approach. Specifically, 100 sets of time profiles were randomly generated from a Gaussian distribution using the measured concentration data as the mean values. The variance of the data noise was estimated from the residuals of the data smoothing procedure. For each dataset, the same data smoothing and slope calculation were performed and the corresponding parameter estimates were obtained by minimizing the error function (see below). The confidence bound was directly estimated from the set of 100 values of Φ. For example, the 95% upper confidence bound of the upper bound of the error function is approximated by the *5*-th largest Φ in this set.

### 4.4. Ensemble Modeling Procedure

In the examples, the error function Φ was set to be:

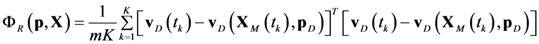
(12)
where *K* is the total number of measurement time points. This error function is implemented in the last step of the incremental identification, where the dependent parameters **p***_D_* are regressed from the dynamic flux estimates **v***_D_*(*t_k_*). Note that the calculation of this error function was actually done one flux at a time, as the least square regression of **p***_D_* from **v***_D_*(*t_k_*) was performed for each flux function separately. For power-laws, this regression can be performed very efficiently, as the logarithm of the flux function depends linearly on the parameters (leading to a linear least square regression). In this case, **v***_D_*(*t_k_*) was calculated from **v***_I_*(*t_k_*) according to Equation (8), while **v***_I_*(*t_k_*) was computed from the time series data and **p***_I_* using the flux function **v***_I_*(**X***_M_*(*t_k_*), **p***_I_*). In other words, the error function depends only on the independent parameters **p*_I_***. The initial parameter point **p***_I_* for the OEAMC algorithm was obtained from the following optimization:

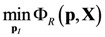
(13)
subject to the bounds on the parameters and fluxes as discussed in the previous section. Other error functions can also be used, for example, using the slope prediction error:

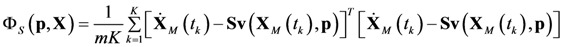
(14)
or the concentration prediction error:

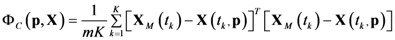
(15)
where **X**(*t_k_*,**p**) is the concentration simulation.

The model ensemble procedure starts with finding an initial viable point for the OEAMC algorithm, as discussed above. Next, the upper bound for the error function will be set either by applying standard statistical analysis assuming Gaussian noise or using the Monte Carlo algorithm described in the previous subsection. The OEAMC is then applied to generate the coarse-grained set of viable parameters over the space of the independent parameters. Finally, this set becomes the input to the MEBS algorithm, producing a population of viable parameters **p***_I_* that represents the ensemble of models. Note that while this work concerns with power-law fluxes, the ensemble generation procedure has general applicability to any kinetic models that can be written as **Ẋ**(*t*, **p**) = **Sv**(**X**, **p**).

## 5. Conclusions

The kinetic modeling of metabolic networks is challenging, but critical in many applications of metabolic engineering. Particularly, parameter identifiability issue, wherein not all parameters can be uniquely determined from the data, has been identified as a common root cause of the difficulty in this process. This uncertainty in parameters implies that there exist (infinitely) many models that will give statistically equivalent goodness of fit to data. Built on the concept of incremental identification, we have proposed an efficient ensemble modeling procedure that relies on three components: (1) data smoothing and approximation of time-series metabolic concentration data, (2) a compact parameter space defining the model ensemble, and (3) efficient parameter exploration. The applications for the ensemble modeling of a generic branched pathway and the trehalose pathway in *Saccharomyces cerevisiae* demonstrate the efficacy of the proposed method.

## Supplementary Files

Supplementary File 1 Supplementary File (DOCX, 242 KB)
