# Supplementary material for: Ensemble Kinetic Modeling of Metabolic Networks from Dynamic Metabolic Profiles
Source: Metabolites. 2012 Nov 12;2(4):891–912. doi: 10.3390/metabo2040891 (PMC3901226; doi:10.3390/metabo2040891)
Supplement: Supplementary File 1 — Supplementary File (DOCX, 242 KB) [file metabolites-02-00891-s001.docx]

Supplementary Material

Ensemble Kinetic Modeling of Metabolic Networks from Dynamic Metabolic Profiles

Gengjie Jia, Gregory Stephanopoulos and Rudiyanto Gunawan

The model parameters used in the generation of *in silico* metabolite time profiles are reported in Table S1.

**Table S1.** True parameter values of the branched pathway model.

| Parameters | Reported Values [[2](#_ENREF_2)] |
| --- | --- |
|  |  |
| *γ_1_* | 20 |
| *f_13_* | 0.8 |
| *γ_2_* | 8 |
| *f_21_* | 0.5 |
| *γ_3_* | 3 |
| *f_32_* | 0.75 |
| *γ_4_* | 5 |
| *f_43_* | 0.5 |
| *f_44_* | 0.2 |
| *γ_5_* | 2 |
| *f_51_* | 0.5 |
| *γ_6_* | 6 |
| *f_64_* | 0.8 |

Following the same ensemble modeling procedure described in the main context, the parameter estimation minimizing slope prediction error Φ*_S_* (Equation (12) in the main context) was conducted to provide an input to the OEAMC algorithm (minimum $\sqrt{\Phi_{S}}$ = 0.137). The upper 95% confidence bound for $\sqrt{\Phi_{S}}$ was estimated to be 0.295.

Table S2 provides the summary of the ensemble construction based on the slope error function Φ*_S_*. The volume of the viable subspace of **p***_I_* was 0.270% of the volume of the original parameter space. The range of values for the slope and concentration errors were again computed from uniformly sampling parameter points from the viable space (*n* = 75680). Figure S1 shows the projections of the viable regions onto the two-dimensional parameter axes of the independent fluxes *v_1_* and *v_6_*. The true parameter values belong to the viable subspace (red dot in Figure S1). Lastly, Figure S2 compares the metabolite concentration predictions produced by five randomly selected member models and the *in silico* generated noisy data used for the construction of the model ensemble. Again, these models could provide similar goodness-of-fit to the data.

**Table S2.** Ensemble kinetic modeling of the branched pathway model using Φ*_S_*.

| CPU time (sec) ^a^ | 1865 |
| --- | --- |
| Calculated volume of initial parameter space  (*V_ci_*) ^b^ | 2.5×10^5^ |
| Estimated volume of viable parameter space  (*V_ev_*) ^c^ | 675.3 ± 4.2 |
| Ratio of *V_ev_* to *V_ci_* | (270.1 ± 1.7) ×10^-3^ % |
| Value range of slope errors $\sqrt{\Phi_{S}}^{\begin{matrix} & d \end{matrix}}$ | [1.370×10^-1^, 2.952×10^-1^] |
| Value range of concentration errors $\sqrt{\Phi_{C}}^{\begin{matrix} & e \end{matrix}}$ | [3.526×10^-2^, 2.366×10^-1^] |

1. The CPU time was the total time for the ensemble construction, which was run on a computer workstation with Dual Processors Intel Quad-Core 2.83 GHz.
2. *V_ci_* was calculated by simple multiplications of the independent parameter ranges.
3. *V_ev_* was calculated by integrating the volumes of an ensemble of ellipsoids that cover the viable parameter space [[1](#_ENREF_1)].
4. The range of slope error was computed using Equation (12) for all models in the ensemble.
5. The range of concentration error was computed by Equation (13) for all models in the ensemble.

**Figure S1.** Two-dimensional projections of the viable parameter space onto the parameter axes of each independent flux (*v_1_*: left, *v_6_*: right). The true parameters are marked in red.


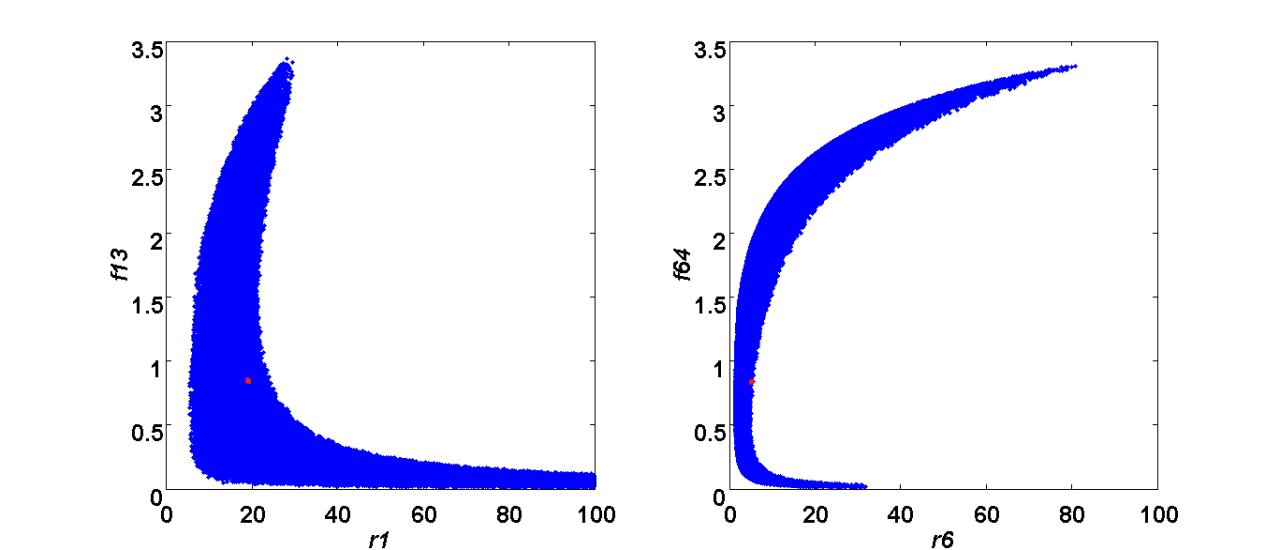


**Figure S2.** Concentration simulations of five randomly selected models from the ensemble (solid blue, brown, green, red and purple lines) versus the noisy data (**×**).

References

1. Zamora-Sillero, E.; Hafner, M.; Ibig, A.; Stelling, J.; Wagner, A., Efficient characterization of high-dimensional parameter spaces for systems biology. *Bmc Systems Biology* **2011**, *5*.

2. Voit, E.O.; Almeida, J., Decoupling dynamical systems for pathway identification from metabolic profiles. *Bioinformatics* **2004**, *20*, 1670-1681.
